# Ballroom Dancing Promotes Neural Activity in the Sensorimotor System: A Resting-State fMRI Study

**DOI:** 10.1155/2018/2024835

**Published:** 2018-04-26

**Authors:** Yingzhi Lu, Qi Zhao, Yingying Wang, Chenglin Zhou

**Affiliations:** School of Kinesiology, Shanghai University of Sport, Shanghai, China

## Abstract

**Objective:**

This study aims at investigating differences in the spontaneous brain activity and functional connectivity in the sensorimotor system between ballroom dancers and nondancers, to further support the functional alteration in people with expertise.

**Materials and Methods:**

Twenty-three ballroom dancers and twenty-one matched novices with no dance experience were recruited in this study. Amplitude of low-frequency fluctuation (ALFF) and seed-based functional connectivity, as methods for assessing resting-state functional magnetic resonance imaging (rs-fMRI) data, were used to reveal the resting-state brain function in these participants.

**Results:**

Compared to the novices, ballroom dancers showed increased ALFF in the left middle temporal gyrus, bilateral precentral gyrus, bilateral inferior frontal gyrus, left postcentral gyrus, left inferior temporal gyrus, right middle occipital gyrus, right superior temporal gyrus, and left middle frontal gyrus. The ballroom dancers also demonstrated lower ALFF in the left lingual gyrus and altered functional connectivity between the inferior frontal gyrus and temporal, parietal regions.

**Conclusions:**

Our results indicated that ballroom dancers showed elevated neural activity in sensorimotor regions relative to novices and functional alterations in frontal-temporal and frontal-parietal connectivity, which may reflect specific training experience related to ballroom dancing, including high-capacity action perception, attentional control, and movement adjustment.

## 1. Introduction

In recent years, with the increase in the number of available exercise options, dance-based exercise, such as ballroom dancing, has become very popular in China. Similar to other traditional dancing forms (e.g., ballet), ballroom dancing requires the synchronization of various body movements according to auditory stimuli. Furthermore, ballroom dancing also demands a high-level domain-specific motor skill. As such, ballroom dancers provide a unique model to investigate how the brain integrates movement and sound and to develop motor expertise combining artistic creativity and performance. A large amount of evidence indicates that motor skill training, including dance, can improve brain function and promote brain plasticity [1–3]. However, to our knowledge, no work has been done to specifically investigate whether ballroom dancing also alters functional plasticity in the brain, especially in sensorimotor areas.

At present, studies on functional brain plasticity related to dance and motor skill mostly focus on task-related functional magnetic resonance imaging (fMRI). For example, the brain activity recorded from professional dancers during the process of observing specific actions indicated increased activity in primary somatosensory cortices, supplementary motor areas, primary motor cortex, the superior parietal lobe, and the inferior parietal lobule [4, 5], areas which may be involved in processes such as gestural motor control, auditory perception, and other aspects of cognition such as emotion and memory [6–8] which are relevant to dance performance. Meanwhile, an investigation featuring professional basketball athletes showed higher activity in inferior parietal lobule and inferior frontal gyrus relative to novices during action anticipation [9]. Taken together, these findings suggest that cognitive-motor activity can induce brain changes (i.e., plasticity) in sensorimotor areas, which may be dependent on the specific motor and sensory processes involved in skill acquisition over time. However, findings from these task-related fMRI studies are contingent upon the task demands and specific paradigms used in the studies, which makes the comparison of results across studies difficult.

Recently, resting-state fMRI (rs-fMRI) has been widely used to study spontaneous brain activity through blood oxygen level dependence (BOLD), without any stimulation or explicit cognitive tasks [10]. Since rs-fMRI does not involve any particular motor or sensory task requirements, it may reflect a cumulative effect of specific experience over time [11]. There are several measurements depicting local features of the BOLD signal. Biswal et al. first reported that spontaneous low-frequency (0.01–0.08 Hz) fluctuations (LFFs) in fMRI were highly synchronous between the right and left primary motor cortices at rest [10]. The functional connectivity patterns of LFFs were quite similar to the activation pattern obtained from a bilateral finger-tapping task, suggesting that LFFs might contain physiologically meaningful information [12]. Further, Zang et al. developed an index, the amplitude of LFFs (ALFF), in which the square root of the power spectrum was integrated in a low-frequency range, for detecting the regional intensity of spontaneous fluctuations in the BOLD signal, which was used in previous rs-fMRI studies with long-term training [13]. For example, Di et al. found that badminton training is associated with greater ALFF in the right and medial cerebellar regions and smaller ALFF in the left superior parietal lobule, indicating the functional alterations in the frontoparietal network for badminton experts [14]. Additionally, a higher ALFF for acupuncturists was found in the left ventral medial prefrontal cortex (VMPFC) and the contralateral hand representation of the primary somatosensory area (SI), compared with the controls, showing the resting-state activity alteration from the expertise-related effects [15]. As such, these studies indicated that cognitive-motor activity can affect the baseline brain activity, as indicated by ALFF, during rest.

Furthermore, functional connectivity also was examined to investigate the functional brain changes associated with expertise. A recent fMRI study assessed the functional connectivity density (FCD) of dancers during rest and reported functional changes in motor regions, especially within sensorimotor cortices, showing higher resting-state functional connectivity density (rs-FCD) in corticobasal loops [16]. Indeed, sensorimotor areas, including the middle cingulate cortex, the bilateral putamen, and the precentral and postcentral gyri, are often reported to be active during watching the dancing performance [17]. However, the brain activity of dancers, specifically ballroom dancers, who have received specific musical and motor training, during a resting state remains largely unknown.

In the current study, we examined the resting-state functional brain activity in professional ballroom dancers, especially in sensorimotor areas. To the best of our knowledge, the functional characteristics of ballroom dancers' brains during rest have not been explored using neuroimaging methods. Similar to professional dancers and other athletes, ballroom dancers require complex skills involving the simultaneous perception of the auditory, visual, and somatosensory modalities. As such, areas underlying these processes may exhibit differences in activation relative to nondancers. Also, given the high degree of motor skill required to process the complex movements, the sensorimotor system may also exhibit differences [18, 19].

We predicted that professional ballroom dancers would exhibit a higher level of spontaneous activity in these sensory and motor regions compared with novices. Furthermore, we also predicted that the functional connectivity among these regions would differ between ballroom dancers and novices. As such, a group of professional ballroom dancers and matched nondancer controls (i.e., novices) were recruited in the present study. The ALFF of rs-fMRI was used to measure regional properties of the brain's intrinsic neural activity and compared between the professional ballroom dancers and controls. Also, based on the between-group regional differences found in the ALFF analysis, functional connectivity analyses of the rs-fMRI were conducted to characterize functional integration.

## 2. Materials and Methods

### 2.1. Participants

Twenty-three female professional ballroom dancers (ages 18–23) were recruited from the Shanghai University of Sport and the Nanjing Sport Institute and served as the dancer group; twenty-one females (ages 19–22) without any dance experience were recruited as the control group. All were right-hand dominant and had no history of neurological disorders. Participants were paid for their participation and signed a consent form before taking part in the study. This study was approved by the ethics committee of at the Center for Cognition and Brain Disorders of the Hangzhou Normal University. The demographic data of the participants are listed in Table 1.

### 2.2. Expertise-Level Criterion

The dancers were recruited from ballroom dance teams and met all the following inclusion criteria: (1) must have 6 or more years of professional training experience; (2) must practice more than three days a week and 2 more hours each practice session during the last 3 years; (3) had attended national ballroom dance competitions in the recent three years; and (4) did not take part in extra physical or sports-relevant training. Controls were recruited from Hangzhou Normal University, did not have any previous experience with dance, and did not consume dancing-related media at all.

### 2.3. MRI Data Acquisition

All images were acquired on a 3T MRI scanner (GE MR750) at the Center for Cognition and Brain Disorders of the Hangzhou Normal University. Before the experiment, all participants were told that this task involved observing ballroom dance video and judging the performance. Before the task, there was a resting data collection session. All participants were required to lie in the MRI scanner, eyes open, viewing a cross on the screen to prevent falling asleep, relax, and think nothing specific. When the resting scan finished, the MRI operator talked with the participant via microphone, to make sure participants remained awake or did not experience discomfort during the scan. All participants reported being awake and gave positive feedback. All the rs-fMRI data were collected before the task-related experiment using a gradient-echo echo-planar imaging sequence (repetition time [TR]/echo time [TE] = 2000/30 ms, flip angle (FA) = 90°, matrix = 64 × 64, field of view (FOV) = 220 × 220 mm^2^, thickness/gap = 3.2/0 mm, and 43 slices). The scan lasted 8 minutes. Subsequently, the magnetization-prepared rapidly acquired gradient-echo T1-weighted scans were obtained (TR/TE = 8.156/3.18 ms, FA = 8°, matrix = 256 × 256, FOV = 256 × 256 mm^2^, and thickness/gap = 1/0mm).

### 2.4. Data Preprocessing

MRI data were processed using dpabi (http://rfmri.org/dpabi) [20]. The processing steps included the removal of the first ten volumes, slice timing, head-motion correction (all participants displayed head-motion in translation < 2 mm and rotation < 2°; no one was excluded), coregistration of the structural data, spatial normalization to the Montreal Neurological Institute (MNI) space T1 Template, and resample into 3 × 3 × 3 mm^3^. Removal of covariates included regression of linear trend, white matter nuisance signals, cerebral spinal fluid BOLD signal, and Friston 24 head motion. Finally, spatial smoothing was conducted with an isotropic Gaussian kernel of 4 mm of full width at half maximum (FWHM).

### 2.5. ALFF Analysis

After data preprocessing, ALFF maps were acquired as follows. For a given voxel, a fast Fourier transform (FFT) was used to convert the filtered time series to a frequency domain to obtain the power spectrum. The power spectrum was then square-rooted and averaged across 0.01–0.1 Hz at each voxel, which was deemed as the ALFF [13]. Then, ALFF was standardized by dividing the whole brain voxel average ALFF, turning into mALFF maps.

### 2.6. Functional Connectivity Analysis

After data preprocessing, a temporal band-pass filter (0.01–0.10 Hz) was applied to reduce low-frequency drift and physiological high-frequency noise. Based on the ALFF results, 10 spherical regions (radius 10 mm) were selected as regions of interest (ROIs). The mean BOLD signal intensity time series was extracted from the ROIs. Subsequently, functional connectivity analysis was performed between the ROIs and all voxels in the brain data through the Pearson correlation coefficient.

### 2.7. Statistical Analysis

To explore the ALFF and functional connectivity differences between the dancer group and the control group, two-sample *t*-tests were performed on standardized mALFF and functional connectivity maps, respectively. Gaussian random field (GRF) theory (voxel significance *P* < 0.001 and cluster significance *P* < 0.010) was used for multiple comparison correction.

## 3. Results

### 3.1. ALFF

**Figure 1:**
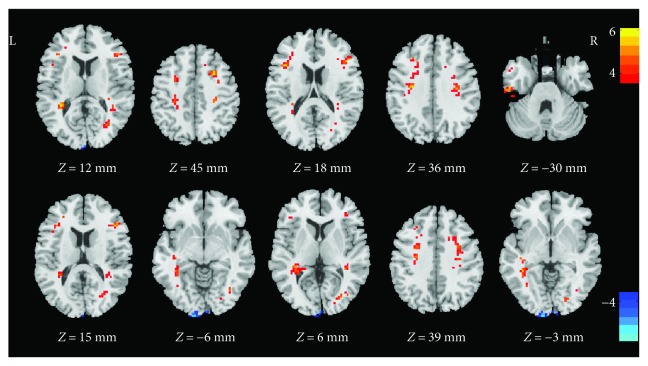
Regions of ALFF differences between the dancer group and the control group. The color bar indicates the *t-*values. The yellow to orange color means the positive values (dancer group minus control group), and the dark to light blue color means the negative values (dancer group minus control group). Clusters with *P* < 0.01 (GRF corrected) and a spatial extent *k* > 20 voxels were considered statistically significant.

Compared to the controls, the dancers showed significantly higher ALFF in the left middle temporal gyrus, bilateral precentral gyrus, bilateral inferior frontal gyrus, left postcentral gyrus, left inferior temporal gyrus, right middle occipital gyrus, right superior temporal gyrus, and left middle frontal gyrus, and less ALFF in left lingual gyrus (Table 2, Figure 1).

### 3.2. Functional Connectivity

**Figure 2:**
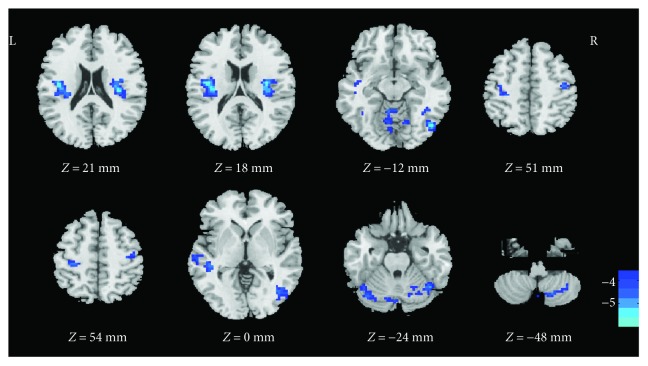
Regions showing a different functional connectivity between the dancer group and the control group when the inferior frontal gyrus was used as the seed. The color bar indicates the *t*-values. The dark to light blue color means the negative values (dancer group minus control group). Clusters with *P* < 0.01 (GRF corrected) and a spatial extent *k* > 20 voxels were considered statistically significant.

Functional connectivity analysis for all participants revealed that seeding regions belonged to distinct functional networks. Since our purpose is to discuss the sensorimotor system, we selected the most relevant result here, that the seed peaked at [−42,18,18] was chosen and presented as follows; other seed results are reported in the supplement material (Supplement 1). Compared to the control group, the dancer group showed that the seed belonging to the inferior frontal gyrus had significantly lower functional connectivity to the bilateral insula, right inferior temporal gyrus, bilateral precentral gyrus, left postcentral gyrus, left middle temporal gyrus, left fusiform gyrus, and right cerebellum (Table 3, Figure 2).

## 4. Discussion

In the current study, we compared the neural activity and functional connectivity among the sensorimotor cortices in professional ballroom dancers and controls, using resting-state fMRI. Two measurements, ALFF and functional connectivity, were assessed in the current study. First, the dancers showed higher ALFF in the left middle temporal gyrus, bilateral precentral gyrus, bilateral inferior frontal gyrus, left postcentral gyrus, left inferior temporal gyrus, right middle occipital gyrus, right superior temporal gyrus, and left middle frontal gyrus compared to the control group. These brain areas mostly belong to the sensorimotor system and correspond to perception, movement control, and other related functions. Second, the dancer group showed lower ALFF in the left lingual gyrus and lower functional connectivity between the inferior frontal gyrus and temporal, parietal regions.

The greater ALFF in the postcentral gyrus, temporal lobe, and middle occipital gyrus in ballroom dancers is consistent with other studies [14, 21]. Postcentral gyrus, located in the primary somatosensory cortex, is part of the action observation and action imitation networks [22] and receives a large number of sensory inputs and storing perceptual experiences [23, 24]. Dancers observe and remember dance movements and imitate and practice with music continually to enhance their motor skills. An important step in the imitation of dance movements is observation. Dancers form a complex action model from an observation, which can be adjusted and implemented by transforming visual information into motor commands. Meanwhile, the temporal lobe and the middle occipital gyrus are involved in audiovisual processing and memory [25–28]. The storage of action information could have affected dancers' handling of the action process when they observed familiar dance movements [29]. Researchers have found that watching videos of dance movements produced significantly higher brain activity in the middle occipital gyrus compared to watching still pictures [30]. Furthermore, activity in the superior temporal gyrus has been associated with the coherence of dance movements and is proportional to the strength of the action connection [31]. Therefore, the current study supports the modulation of ballroom dance training on the sensory regions.

Besides these sensory input regions, we also found increased ALFF in the precentral gyri which belong to the primary motor cortex [32] and have been related to motor performance, including action memory [33], motor skill learning [34, 35], and motor control [36, 37]. This finding may reflect ballroom dancers' greater engagement of action memory systems during action observation, adjusting the spatial orientation, speed, melody, and amplitude of the actions, altering precentral gyrus activation relative to novices. Moreover, a previous study revealed that the precentral gyri were related to the degree of movement mastery [5], as activation in the precentral gyri was significantly increased over five weeks of dancing practice. Thus, the present findings may reflect the brain plasticity after long-term ballroom dancing training. However, further confirmation should be used by a longitudinal design in the future.

Additionally, the middle and inferior frontal gyri also are involved in attention control [38, 39]. The increased activity in these regions among the ballroom dancers may imply that dancers are able to apply a greater focus attention to dance sequence, in order to enhance the movement skills, which may be related to their greater perceptual and movement control capabilities as explained earlier, possibly due to extensive practice [40, 41].

However, we also observed a reduced ALFF in lingual gyrus and reduced functional connectivity in the frontal-parietal and frontal-temporal networks. The lingual gyrus is linked to visual processing, especially related to letters, which we speculate was due to the extensive professional motor and musical training received by the dancers. The observed lower functional connectivity appears to be inconsistent with previous findings featuring musicians which revealed increased rs-FC between motor and multisensory cortices (such as visual, auditory, and somatosensory cortices) relative to novices [42]. However, the current result is partially consistent with other studies featuring dancers and athletes. Previous research reported an increased rs-FC between the right supramarginal gyrus and right precentral gyrus after an initial 2 weeks of training, but found a decreased rs-FC in the last two weeks when the behavioral performance started to improve and stabilize [43]. A similar decrease in connectivity was also found among expert badminton players [14]. Therefore, it may be that the decreased connectivity in the dancer group between frontal-central and frontal-temporal regions are associated with a reduction of attentive load and an automation of motor skills [44]. Further longitudinal studies are needed to better interpret the directionality of functional connectivity in different brain regions and in different motor learning stages.

The above result may also support the notion of dance as a treatment for certain motor disorders. Previous studies have used dance-based interventions to improve balance during gait and joint mobility at the physical level in patients with Parkinson's disease [45]. Indeed, neuroimaging studies revealed that dance lessons can stimulate activation of the premotor and supplementary motor areas [46]. Additionally, it has also been reported that dance-based exercise improved memory and executive function and increased participation in complex daily activities in Parkinson patients [47, 48]. While the mechanism of these changes is unclear, functional modulation of brain resting state may be involved. Further studies are needed to investigate this point.

Finally, there are several limitations in the present study which should be noted. First, the relative sample size is small, all participants are females [49], and some potential confounds, such as physical activity levels [50], were not assessed. Further research should be mindful of these issues. Secondly, the present study is a cross-sectional design; a longitudinal design would be more revealing as to the potential mechanisms involved in the observed brain changes.

## 5. Conclusions

In the current study, we indicated the differences in ALFF and functional connectivity between ballroom dancers and novices, which provided new evidence that ballroom dancing can alter the function of the sensorimotor system. According to the present data, select perceptual and motor neurological functions appear to be promoted in the ballroom dancers compared to novices, providing further evidence that ballroom dancing, a unique form of physical activity, might be related to cortical plasticity of the sensorimotor system.

## Figures and Tables

**Table 1:** The demographic data of the dancer group and control group.

	Dancer group	Control group	*P* value
Age (years)	20.83 ± 1.56	20.82 ± 0.81	>0.05
Education (years)	14.96 ± 0.83	15.10 ± 0.44	>0.05
BMI (kg/m^2^)	19.43 ± 1.52	18.56 ± 1.24	>0.05
Years of training	9.00 ± 3.33	0	<0.01
Frequency (times/week)	2–6	0	<0.01
Duration (hours)	1–3	0	<0.01

Note: BMI: body mass index; data are expressed as mean values ± standard deviation; *P* values were derived from Student's *t*-test comparing the two groups. The subtype of the ballroom dance includes rumba, samba, cha-cha, jive, and pasodoble.

**Table 2:** Brain regions with significantly different ALFF values between the dancer group and the control group.

Brain regions	Side	BA	MNI coordinates			Cluster size	*t*-value
			x	y	z		
Middle temporal gyrus	L	21	−33	−42	12	265	6.14
Precentral gyrus	R	6/8	27	12	45	161	6.11
Inferior frontal gyrus	L	48	−42	18	18	72	5.69
Precentral gyrus	L	6	−30	−12	36	95	5.56
Postcentral gyrus	L	3	−30	−12	36	95	5.56
Inferior temporal gyrus	L	20	−51	−18	−30	99	5.43
Inferior frontal gyrus	R	45	45	27	15	37	5.37
Middle occipital gyrus	R	19	36	−69	−6	66	4.79
Superior temporal gyrus	R	37	36	−39	6	28	4.77
Middle frontal gyrus	L	6	−27	3	39	31	4.71
Lingual gyrus	L	17	−12	−105	−3	107	−5.80

Note: BA: Brodmann area; MNI: Montreal Neurological Institute; L: left; R: right.

**Table 3:** Brain regions with significantly different functional connectivity values between the dancer group and the control group.

Brain regions	Side	BA	MNI coordinates			Cluster size	*t*-value
			x	y	z		
Insula	L	13	−42	−12	21	117	−5.98
Insula	R	13	33	−9	18	118	−5.96
Inferior temporal gyrus	R	37	48	−69	−12	496	−5.92
Precentral gyrus	R	4	42	−15	51	72	−4.74
Precentral gyrus	L	4	−27	−27	54	83	−4.61
Postcentral gyrus	L	4	−27	−27	54	83	−4.61
Middle temporal gyrus	L	22	−42	−33	0	118	−4.61
Fusiform gyrus	L	37	−36	−69	−24	63	−4.54
Cerebellum	R		18	−66	−48	72	−4.51

Note: BA: Brodmann area; MNI: Montreal Neurological Institute; L: left; R: right.

## Supplementary Materials

Supplementary Materials Supplement 1: since our purpose is to discuss the sensorimotor system, we selected the most relevant result to report in the Results section, and the other seed functional connectivity results are reported as follows. Brain regions with significantly different functional connectivity values between the dance group and the control group.

## References

[1] Cotman C. W., Berchtold N. C. (2002). Exercise: a behavioral intervention to enhance brain health and plasticity. *Trends in Neurosciences*.

[2] Green C. S., Bavelier D. (2008). Exercising your brain: a review of human brain plasticity and training-induced learning. *Psychology and Aging*.

[3] Voss M. W., Vivar C., Kramer A. F., van Praag H. (2013). Bridging animal and human models of exercise-induced brain plasticity. *Trends in Cognitive Sciences*.

[4] Calvo-Merino B., Glaser D. E., Grèzes J., Passingham R. E., Haggard P. (2005). Action observation and acquired motor skills: an FMRI study with expert dancers. *Cerebral Cortex*.

[5] Cross E. S., Hamilton A. F. D. C., Grafton S. T. (2006). Building a motor simulation de novo: observation of dance by dancers. *NeuroImage*.

[6] Koelsch S., Siebel W. A. (2005). Towards a neural basis of music perception. *Trends in Cognitive Sciences*.

[7] Levitin D. J., Tirovolas A. K. (2009). Current advances in the cognitive neuroscience of music. *Annals of the New York Academy of Sciences*.

[8] Hänggi J., Koeneke S., Bezzola L., Jäncke L. (2010). Structural neuroplasticity in the sensorimotor network of professional female ballet dancers. *Human Brain Mapping*.

[9] Wu Y., Zeng Y., Zhang L. (2013). The role of visual perception in action anticipation in basketball athletes. *Neuroscience*.

[10] Biswal B., Zerrin Yetkin F., Haughton V. M., Hyde J. S. (1995). Functional connectivity in the motor cortex of resting human brain using echo-planar MRI. *Magnetic Resonance in Medicine*.

[11] Lewis C. M., Baldassarre A., Committeri G., Romani G. L., Corbetta M. (2009). Learning sculpts the spontaneous activity of the resting human brain. *Proceedings of the National Academy of Sciences*.

[12] Zou Q. H., Zhu C. Z., Yang Y. (2008). An improved approach to detection of amplitude of low-frequency fluctuation (ALFF) for resting-state fMRI: fractional ALFF. *Journal of Neuroscience Methods*.

[13] Zang Y. F., He Y., Zhu C. Z. (2007). Altered baseline brain activity in children with ADHD revealed by resting-state functional MRI. *Brain & Development*.

[14] Di X., Zhu S., Jin H. (2012). Altered resting brain function and structure in professional badminton players. *Brain Connectivity*.

[15] Dong M., Li J., Shi X. (2015). Altered baseline brain activity in experts measured by amplitude of low frequency fluctuations (ALFF): a resting state fMRI study using expertise model of acupuncturists. *Frontiers in Human Neuroscience*.

[16] Li G., He H., Huang M. (2015). Identifying enhanced cortico-basal ganglia loops associated with prolonged dance training. *Scientific Reports*.

[17] Karpati F. J., Giacosa C., Foster N. E. V., Penhune V. B., Hyde K. L. (2015). Dance and the brain: a review. *Annals of the New York Academy of Sciences*.

[18] Riemann B. L., Lephart S. M. (2002). The sensorimotor system, part I: the physiologic basis of functional joint stability. *Journal of Athletic Training*.

[19] Riemann B. L., Lephart S. M. (2002). The sensorimotor system, part II : the role of proprioception in motor control and functional joint stability. *Journal of Athletic Training*.

[20] Yan C. G., Wang X. D., Zuo X. N., Zang Y. F. (2016). DPABI: data processing & analysis for (resting-state) brain imaging. *Neuroinformatics*.

[21] Kim J. H., Han J. K., Kim B. N., Han D. H. (2015). Brain networks governing the golf swing in professional golfers. *Journal of Sports Sciences*.

[22] Caspers S., Zilles K., Laird A. R., Eickhoff S. B. (2010). ALE meta-analysis of action observation and imitation in the human brain. *NeuroImage*.

[23] Powell T. P., Mountcastle V. B. (1959). Some aspects of the functional organization of the cortex of the post central gyrus of the monkey: a correlation of findings obtained in a single unit analysis with cytoarchitecture. *Bulletin of the Johns Hopkins Hospital*.

[24] Jenkins W. M., Merzenich M. M., Ochs M. T., Allard T., Guíc-Robles E. (1990). Functional reorganization of primary somatosensory cortex in adult owl monkeys after behaviorally controlled tactile stimulation. *Journal of Neurophysiology*.

[25] McCarley R. W., Shenton M. E., O'Donnell B. F. (1993). Auditory P300 abnormalities and left posterior superior temporal gyrus volume reduction in schizophrenia. *Archives of General Psychiatry*.

[26] Squire L., Zola-Morgan S. (1991). The medial temporal lobe memory system. *Science*.

[27] Riches I. P., Wilson F. A., Brown M. W. (1991). The effects of visual stimulation and memory on neurons of the hippocampal formation and the neighboring parahippocampal gyrus and inferior temporal cortex of the primate. *The Journal of Neuroscience*.

[28] Vandenberghe R., Price C., Wise R., Josephs O., Frackowiak R. S. J. (1996). Functional anatomy of a common semantic system for words and pictures. *Nature*.

[29] Calvo-Merino B., Ehrenberg S., Leung D., Haggard P. (2010). Experts see it all: configural effects in action observation. *Psychological Research PRPF*.

[30] Cross E. S., Kirsch L., Ticini L. F., Schütz-Bosbach S. (2011). The impact of aesthetic evaluation and physical ability on dance perception. *Frontiers in Human Neuroscience*.

[31] Bachrach A., Jola C., Pallier C. (2016). Neuronal bases of structural coherence in contemporary dance observation. *NeuroImage*.

[32] Mushiake H., Inase M., Tanji J. (1991). Neuronal activity in the primate premotor, supplementary, and precentral motor cortex during visually guided and internally determined sequential movements. *Journal of Neurophysiology*.

[33] Shadmehr R., Holcomb H. H. (1997). Neural correlates of motor memory consolidation. *Science*.

[34] Kami A., Meyer G., Jezzard P., Adams M. M., Turner R., Ungerleider L. G. (1995). Functional MRI evidence for adult motor cortex plasticity during motor skill learning. *Nature*.

[35] Muellbacher W., Ziemann U., Boroojerdi B., Cohen L., Hallett M. (2001). Role of the human motor cortex in rapid motor learning. *Experimental Brain Research*.

[36] Graziano M. S. A., Taylor C. S. R., Moore T. (2002). Complex movements evoked by microstimulation of precentral cortex. *Neuron*.

[37] Lemon R. N. (2008). Descending pathways in motor control. *Annual Review of Neuroscience*.

[38] Hampshire A., Chamberlain S. R., Monti M. M., Duncan J., Owen A. M. (2010). The role of the right inferior frontal gyrus: inhibition and attentional control. *NeuroImage*.

[39] Hopfinger J. B., Buonocore M. H., Mangun G. R. (2000). The neural mechanisms of top-down attentional control. *Nature Neuroscience*.

[40] Kattenstroth J. C., Kalisch T., Kolankowska I., Dinse H. R. (2011). Balance, sensorimotor, and cognitive performance in long-year expert senior ballroom dancers. *Journal of Aging Research*.

[41] Chang M., Halaki M., Adams R., Cobley S., Lee K. Y., O'Dwyer N. (2016). An exploration of the perception of dance and its relation to biomechanical motion: a systematic review and narrative synthesis. *Journal of Dance Medicine & Science*.

[42] Wan C. Y., Schlaug G. (2010). Music making as a tool for promoting brain plasticity across the life span. *The Neuroscientist*.

[43] Ma N., Liu Y., Fu X. M. (2011). Abnormal brain default-mode network functional connectivity in drug addicts. *PLoS One*.

[44] Bezzola L., Mérillat S., Jäncke L. (2012). The effect of leisure activity golf practice on motor imagery: an fMRI study in middle adulthood. *Frontiers in Human Neuroscience*.

[45] Earhart G. M. (2009). Dance as therapy for individuals with Parkinson disease. *European Journal of Physical and Rehabilitation Medicine*.

[46] Sacco K., Cauda F., Cerliani L., Mate D., Duca S., Geminiani G. C. (2006). Motor imagery of walking following training in locomotor attention. The effect of ‘the tango lesson’. *NeuroImage*.

[47] Hashimoto H., Takabatake S., Miyaguchi H., Nakanishi H., Naitou Y. (2015). Effects of dance on motor functions, cognitive functions, and mental symptoms of Parkinson’s disease: a quasi-randomized pilot trial. *Complementary Therapies in Medicine*.

[48] Foster E. R., Golden L., Duncan R. P., Earhart G. M. (2013). Community-based Argentine tango dance program is associated with increased activity participation among individuals with Parkinson’s disease. *Archives of Physical Medicine and Rehabilitation*.

[49] Zhang S., Li C.-s. R. (2012). Functional connectivity mapping of the human precuneus by resting state fMRI. *NeuroImage*.

[50] Erickson K. I., Miller D. L., Weinstein A. M., Akl S. L., Banducci S. (2012). Physical activity and brain plasticity in late adulthood: a conceptual and comprehensive review. *Ageing Research*.

