# Supplementary material for: Ballroom Dancing Promotes Neural Activity in the Sensorimotor System: A Resting-State fMRI Study
Source: Neural Plast. 2018 Apr 26;2018:2024835. doi: 10.1155/2018/2024835 (PMC5944238; doi:10.1155/2018/2024835)
Supplement: Supplementary Materials — Supplement 1: since our purpose is to discuss the sensorimotor system, we selected the most relevant result to report in the Results section, and the other seed functional connectivity results are reported as follows. Brain regions with significantly different functional connectivity values between the dance group and the control group. [file 2024835.f1.pdf]

Functional Connectivity Results

1. the sphere peak at [-33, -42, 12], radium = 10 mm

TABLE: Brain regions with significantly different functional connectivity values between the dance group and control group.

Brain regions	Side	BA	MNI coordinates			Cluster size	*t* value
			x	y	z		
Inferior parietal gyrus	L	7	-21	-54	36	72	-4.84
Middle occipital gyrus	L	7	-21	-54	36	72	-4.84

Notes: BA, Brodmann area; MNI, Montreal Neurological Institute; L, left; R, right.

2. the sphere peak at [-51, -18, -30], radium = 10 mm

TABLE: Brain regions with significantly different functional connectivity values between the dance group and control group.

Brain regions	Side	BA	MNI coordinates			Cluster size	*t* value
			x	y	z		
Superior frontal gyrus	L	6	-15	3	75	61	-5.46
Middle occipital gyrus	L	37	-45	-51	-21	117	-5.41
Postcentral gyrus	R	2	15	-39	75	69	-5.11
Rolandic operculum gyrus	R	42	69	-12	15	73	-4.77

Notes: BA, Brodmann area; MNI, Montreal Neurological Institute; L, left; R, right.

3. the sphere peak at [45, 27, 15], radium = 10 mm

TABLE: Brain regions with significantly different functional connectivity values between the dance group and control group.

Brain regions	Side	BA	MNI coordinates			Cluster size	*t* value
			x	y	z		
Superior parietal gyrus	L	7	-24	-54	54	80	-6.42
Middle occipital gyrus	L	19	-39	-57	-9	977	-5.88
Fusiform gyrus	L	37	-39	-57	-9	977	-5.88
cerebellum	L	37	-39	-57	-9	977	-5.88
Fusiform gyrus	R	37	-39	-57	-9	977	-5.88
cerebellum	R	37	-39	-57	-9	977	-5.88
Superior occipital gyrus	R	19	15	-93	27	59	-5.10

Notes: BA, Brodmann area; MNI, Montreal Neurological Institute; L, left; R, right.

4. the sphere peak at [36, -69, -6], radium = 10 mm

TABLE: Brain regions with significantly different functional connectivity values between the dance group and control group.

Brain regions	Side	BA	MNI coordinates			Cluster size	*t* value
			x	y	z		
Insula	R	13	18	-15	24	86	-6.23
Lingual gyrus	R	18	30	-99	-12	249	-5.56
Inferior frontal gyrus	L	47	-48	27	-9	140	-5.55
Medial frontal gyrus	L	9	-3	48	54	205	-5.28
Lingual gyrus	L	18	-24	-96	-15	106	-5.27
Inferior temporal gyrus	L	20	-57	0	-27	90	-5.08
Inferior frontal gyrus	R	47	42	27	-6	78	-5.07
Middle temporal gyrus	R	38	54	12	-33	74	-4.93

Notes: BA, Brodmann area; MNI, Montreal Neurological Institute; L, left; R, right.

5. the sphere peak at [-27, 3, 39], radium = 10 mm

TABLE: Brain regions with significantly different functional connectivity values between the dance group and control group.

Brain regions	Side	BA	MNI coordinates			Cluster size	*t* value
			x	y	z		
Superior frontal gyrus	L	10	-6	60	33	521	-6.76
Medial frontal gyrus	L	10	-6	60	33	521	-6.76
Cerebellum	L	19	-36	-78	-21	956	-5.21
Cerebellum	R	19	-36	-78	-21	956	-5.21

Notes: BA, Brodmann area; MNI, Montreal Neurological Institute; L, left; R, right.
